# Association between Malaria Infection and Early Childhood Development Mediated by Anemia in Rural Kenya

**DOI:** 10.3390/ijerph17030902

**Published:** 2020-02-02

**Authors:** Erin M. Milner, Patricia Kariger, Amy J. Pickering, Christine P. Stewart, Kendra Byrd, Audrie Lin, Gouthami Rao, Beryl Achando, Holly N. Dentz, Clair Null, Lia C.H. Fernald

**Affiliations:** 1 Bureau for Global Health, United States Agency for International Development (USAID), Washington, DC 20024, USA; 2 School of Public Health, University of California, Davis, Davis, CA 95616, USA; patriciakariger@gmail.com; 3 Department of Civil and Environmental Engineering, Tufts University, Medford, MA 02155, USA; amyjanel@gmail.com; 4 Department of Nutrition, University of California, Davis, Davis, CA 95616, USA; cpstewart@ucdavis.edu; 5 WorldFish, Pulau Pinang 11960, Malaysia; K.Byrd@cgiar.org; 6 Division of Epidemiology and Biostatistics, University of California, Berkeley, Berkeley, CA 94720, USA; audrielin@berkeley.edu; 7 Waterborne Disease Prevention Branch, Division of Foodborne, Waterborne, and Environmental Diseases, United States Centers for Disease Control and Prevention, Atlanta, GA 30329, USA; gouthami.g.rao@gmail.com; 8 Innovations for Poverty Action, Nairobi 00200, Kenya; berylachando@gmail.com; 9 Social Impact, Arlington, VA 22201, USA; hollydentz@gmail.com; 10 Mathematica Policy Research, Washington, DC 20002, USA; clair.a.null@gmail.com; 11 Community Health Sciences, University of California, Berkeley, Berkeley, CA 94720, USA; fernald@berkeley.edu

**Keywords:** early childhood development, malaria, anemia, rural Kenya

## Abstract

Malaria is a leading cause of morbidity and mortality among children under five years of age, with most cases occurring in Sub-Saharan Africa. Children in this age group in Africa are at greatest risk worldwide for developmental deficits. There are research gaps in quantifying the risks of mild malaria cases, understanding the pathways linking malaria infection and poor child development, and evaluating the impact of malaria on the development of children under five years. We analyzed the association between malaria infection and gross motor, communication, and personal social development in 592 children age 24 months in rural, western Kenya as part of the WASH Benefits environmental enteric dysfunction sub-study. Eighteen percent of children had malaria, 20% were at risk for gross motor delay, 21% were at risk for communication delay, and 23% were at risk for personal social delay. Having a positive malaria test was associated with increased risk for gross motor, communication, and personal social delay while adjusting for child characteristics, household demographics, study cluster, and intervention treatment arm. Mediation analyses suggested that anemia was a significant mediator in the pathway between malaria infection and risk for gross motor, communication, and personal social development delays. The proportion of the total effect of malaria on the risk of developmental delay that is mediated by anemia across the subscales was small (ranging from 9% of the effect on gross motor development to 16% of the effect on communication development mediated by anemia). Overall, malaria may be associated with short-term developmental delays during a vulnerable period of early life. Therefore, preventative malaria measures and immediate treatment are imperative for children’s optimal development, particularly in light of projections of continued high malaria transmission in Kenya and Africa.

## 1. Introduction

Malaria is a leading cause of morbidity [1] and mortality [2] globally, particularly among children under five years of age [3]. It is an acute febrile illness with an incubation period of seven days or longer. In 2018, more than two-thirds of malaria deaths were among children under five years [4]. Moreover, children under five years have a higher risk of acquiring malaria and are more likely to experience severe adverse effects compared to children over five years [5,6]. The consequences of malaria infection in children include anemia, respiratory distress, and cerebral damage. Over 90% of all malaria cases and deaths occurred in Africa in 2017 [4]. The most common parasite in Africa and the East Africa region (*P. falciparum*) has severe cerebral symptoms associated with it [7]. Malaria is endemic in western Kenya, which has the highest prevalence of *P. falciparum* in the country [7].

Forty-three percent of children under five in low- and middle-income countries (LMICs) are also at risk of not meeting their developmental potential, with children in Africa being at highest risk due to many factors, including extreme poverty [8]. There is growing evidence suggesting that severe malaria is a risk factor for poor child development [9]; however, there are research gaps in quantifying the risk of milder cases of malaria, as most studies assess severe malaria. There are also gaps in understanding the pathway between malaria infection and poor child development, particularly among children under five years. Better understanding of the role of malaria in children’s development is important because developmental delays during the first five years of life can have lifelong implications related to educational attainment, adult wage earning, and health [8]. These long-term adverse effects can be prevented by identifying all benefits of prevention to implement the most cost-effective approach. 

### 1.1. Characteristics of the Association between Malaria and Early Childhood Development 

Developmental deficits related to malaria are complex and varied. In a systematic review of the relation between *P. falciparum* infection and cognitive function, Kihara et al. reported that specific parts of the brain may not always be impacted more than others by malaria as the effects can vary and be diffuse [10]. Impairments are driven by severity of malaria, symptomology, and pre- and post-disease exposure [11]. Malaria infection has been found to be significantly associated with a variety of impairments in areas such as language [12,13], motor [14], memory [14,15,16], executive function [13], and attention [15,16,17] in prospective case-control studies of children in Sub-Saharan Africa. Results vary, however, largely due to heterogeneity in the timing of malaria onset compared with outcome assessments, sample sizes, and measurements of risk factors, malaria infection, and child development outcomes [10,11]. 

The path to cognitive impairment may result from many interacting biological and social risk factors [12]. Risk factors for cognitive impairment associated with malaria include fetal exposure to malaria, repeated malaria infections, malaria symptoms (seizures, coma duration, psychosis, impaired consciousness, hypoglycemia, high fever, inflammation, lethargy, and compromised play/learning time), antimalarial medication, malnutrition, and low socioeconomic status [5,18,19]. In endemic areas where children are prone to repeated infections and other risk factors, malaria can be considered a syndrome with enhanced longer term brain and behavior effects [11].

The functions that are impaired may depend on the stage of brain growth at which the infection is acquired and impairment may not manifest until a child is older [5]. Age at malaria infection is a critical factor influencing cognitive impairment [5,6] and the pathways connecting malaria and child development change with age [20,21,22,23]. Although most research is on school-age children, malaria attacks are more common and severe in younger children and cognitive effects might be worse in children under five years of age [6,9]. For instance, in cross-sectional studies in Zanzibar, Tanzania, malaria among children under two years was associated with lower motor activity, lower language scores, and later onset of walking controlling for socioeconomic status, length-for-age Z-scores, anemia status, and season [20,24,25]. In Uganda, two longitudinal studies found that 12 months after having malaria, children under five years had low cognitive ability, attention, memory [17], gross motor, fine motor, visual perception, and language scores [14]; however, these studies only include children who had one cerebral malaria episode.

### 1.2. Anemia Mechanism Linking Malaria and Early Childhood Development

Malaria may impact early childhood development through a pathway of anemia/low hemoglobin concentrations. It is well-established that malaria causes anemia, especially in children under five years in Sub-Saharan Africa [26,27,28,29]. Malaria leads to anemia via two pathways: through the rapid destruction of red blood cells and as a result of virus-induced systemic inflammation that triggers a release of the iron-regulatory hormone hepcidin from the liver [26]. Hepcidin effectively blocks the absorption of iron in the intestine and leads to sequestration of iron in macrophages [30]. This hepcidin-induced hypoferremia can lead to iron-deficiency anemia, if prolonged. Recent evidence also highlights the role of hepcidin-mediated effects of inflammation on brain iron metabolism with cognitive implications [31,32,33]. Children 6 to 24 months of age with iron-deficiency anemia are at risk for poorer cognitive, motor, social-emotional, and neurophysiologic development in the short- and long-term [34]. Therefore, iron-deficiency anemia may be an important mechanism in the association between malaria and child development. 

Although there is evidence that iron-deficiency anemia can lead to poor child development, there is a research gap in assessing malaria, anemia, and child development. Higher iron concentrations can increase malaria risk among children [35]. However, iron supplementation can increase hemoglobin concentrations in young children and in the WASH (water, sanitation, and hygiene) Benefits trial in Kenya, lipid-based nutrient supplements (LNS) increased hemoglobin concentrations, reduced anemia, and increased iron status [36]. In a randomized controlled trial of malaria prevention among children under three years of age in Uganda, anemia mediated the negative association between malaria infection and cognitive performance in all treatment arms [37]. In Tanzania, hemoglobin concentrations played a significant role in the pathway between malaria and motor abilities among children as they started to walk [24]. More well-designed studies longitudinally evaluating the pathways (including anemia) between mild and severe malaria infection, and a variety of child development outcomes among children under five years are needed to elucidate causal mechanisms and account for seasonal and age-dependent nuances.

### 1.3. Malaria and Child Health in Kenya

Malaria is widespread throughout Kenya and a leading cause of morbidity and mortality in the country [38]. About 74% of the Kenyan population is at risk for malaria and malaria accounts for 30% to 50% of all outpatient clinic or hospital visits [38]. In western Kenya, malaria is endemic and transmission is perennial with seasonal peaks in May to July and October to November [39]. Malaria is estimated to cause 20% of all deaths in children under age five years in the country [38] and the disease is common in this vulnerable population of young children [40]. Malaria and anemia are concurrently high in many parts of Kenya [41]. In 2016, the prevalence of anemia among children under five years in Kenya was 41% [42]. The prevalence of other indicators of poor child health related to nutrition and child development in Kenya is high. Information about early childhood development in Kenya is limited however, and most of the program and policy efforts in the country focus on preschool-age children despite the importance of development in younger years [43].

### 1.4. Objectives

This study aimed to describe the association between malaria infection and early childhood development. The objectives of the research were to: 1) investigate associations between malaria infection and three child development outcomes, and 2) assess the role of anemia as a potential mediator in the pathway between malaria infection and child development outcomes. The following two connected hypotheses were tested: 1) malaria infection is associated with increased odds of being at risk for gross motor, communication, and personal social developmental delays, and 2) anemia is a significant mediator along the pathway between malaria infection and being at risk for gross motor, communication, and personal social developmental delays.

## 2. Materials and Methods 

### 2.1. Setting and Population

These data were collected during the WASH Benefits trial, which is a cluster-randomized controlled trial assessing the impact of WASH and nutrition interventions individually and combined on child health outcomes [44,45,46]. The study was conducted in Bungoma and Kakamega counties in western Kenya. The region is rural and the majority of the population are subsistence farmers. The WASH Benefits trial took place from November 2012 to July 2016. Households were selected to participate in the trial if they were in a rural community (defined as <25% of residents living in a rented home (most families in rural areas own homes compared with urban households in Kenya), with <2 gas stations, and <10 shops in the area) and had limited access to water and sanitation (defined as communities where >80% of residents did not have access to piped water). Female participants were eligible for enrollment into the study if they were pregnant, if they or their partner owned their house, and if they were not planning to move within the next 12 months. The infant that was in utero during the village census was the index child. 

### 2.2. Study Design

Households were visited at enrollment and two follow-up periods, one and two years later. A sub-study assessing environmental enteric dysfunction, malaria infection, and hemoglobin concentrations from biological samples enrolled 2,304 households from four arms of the WASH Benefits trial (WASH, WASH and Nutrition, Nutrition, or Active Control) [36,44,47]. The nutrition intervention entailed nutrition education and all children between 6–24 months in the participating household receiving LNS with iron. In the second follow-up, 1,449 children were included in the sub-study [36,44,47]. This sample size was calculated based on having the power to detect differences in environmental enteropathy biomarkers [48] and this sample size was sufficient to determine the association between malaria infection and child development outcomes. An equal proportion of children were selected from each intervention arm. 

Caregivers brought study children to a central location for sub-study data collection between August 2015 and April 2016. Blood samples for malaria and anemia diagnoses were taken by trained phlebotomists and health surveys were completed. Caregivers provided written informed consent. Consent for blood collection and a sufficient blood sample was obtained for 699 children and therefore malaria and hemoglobin measurements only existed for this subset of children. An average of two to three months later, caregivers brought study children to a central location for main trial data collection, including a child development assessment. Local enumerators conducted most surveys in Kiswahili, the regional language. Surveys and data collection tools were developed from validated measures [49] and locally adapted.

### 2.3. Malaria and Anemia Data Collection

Venous blood samples were drawn from sub-study participant children in a central location within villages for biomarker analyses and drops of blood were used to assess malaria parasites and hemoglobin concentrations. Rapid diagnostic tests (RDTs) for *P. falciparum, P. vivax, P. ovale,* and *P. malariae* were conducted using rapid diagnostic kits (SD Bioline, Malaria Ag, P.f, P.f, P.v, Alere). The kits can detect parasite concentrations up to 28 days after infection. All children with a positive test result were defined as having malaria in these analyses. Axillary temperatures were taken for all children and if the malaria parasite test was positive and the child’s temperature was 37.5 degrees Celsius or higher, he/she was referred to the nearest clinic for malaria treatment. Hemoglobin concentrations were measured using a Hemocue (Hb 301) machine (Angelholm, Sweden). Anemia was defined as having a hemoglobin concentration below 11.0 g/dL [50]. A medical history form was also completed with caregivers about the child’s recent experiences with illness, prophylaxis, and treatment related to a variety of health conditions, including malaria. 

### 2.4. Socio-Demographics

Household demographics, including asset ownership and maternal education, were assessed using standardized questionnaires administered at enrollment. Socioeconomic status was defined with an asset index using enrollment survey results for all sub-study participants. The asset index was created using the first principal component from a principal components analysis, which has been shown to be an appropriate proxy for household wealth and is correlated with consumption expenditures [51,52,53]. Items included in the asset index were housing structure, electricity, and ownership of a radio, television, mobile phone, clock, bicycle, motorcycle, gas stove, and car. Maternal education was categorized into the following categories: incomplete primary school, completed primary school, and any secondary school.

### 2.5. Early Childhood Development

Three domains of child development were assessed: gross motor, communication, and personal social. The corresponding subscales of the Extended Ages and Stages Questionnaire (EASQ) were used to measure each developmental outcome among all WASH Benefits trial participants during the last data collection round (at approximately age 24 months). The EASQ is a modified version of the Ages and Stages Questionnaire (ASQ), which is widely used for the developmental screening of children under five years of age [54,55,56] and has been used in LMICs [57]. The questionnaires contain a series of age-specific items assessing the achievement of developmental milestones and tracking the child’s progress. The EASQ was piloted to assess respondent bias, maternal accuracy in reporting children’s abilities, cultural appropriateness, and feasibility according to standard procedures [49]. The Gross Motor subscale evaluates body and muscle movement, including tasks like standing, walking, and balancing. The Communication subscale assesses language development and the use of words or sounds to express feelings. The Personal Social subscale reflects emotional responses and social interactions. The EASQ was translated and adapted for this study by using local culturally appropriate items, examples, and tasks, but no substantial changes were made to the original questionnaires. Continuous scores for each subscale were calculated and converted to age-adjusted Z-scores based on the control group within the larger WASH Benefits trial. Children were considered at risk for delay in this population if their Z-scores were below the 25th percentile of control group Z-scores for each domain. This method of defining risk for delay was used as a conservative strategy because there was no prior research informing a culturally appropriate clinical cut-off in this study population. Binary variables were used to incorporate a quantitative definition of poor development and to be consistent with analyses used in the main WASH Benefits publications [46,58].

### 2.6. Statistical Analyses

All statistical analyses were conducted in Stata 14 (StataCorp, College Station, TX, USA, 2015). Three sets of logistic multivariate analyses were undertaken to test the hypotheses corresponding to each child development outcome (gross motor, communication, and personal social). Two regression models were built for each child development outcome to assess the pathways between malaria infection (binary) and being at risk for gross motor, communication, and personal social delays (binary). The first model included malaria infection (binary) and the child development outcomes (three separate binary outcomes). Anemia status (binary; anemia defined as hemoglobin concentration <11.0 g/dL) was added to the second model. The interaction between malaria status and participation in the nutrition intervention was also analyzed in multivariate models to assess whether being in a nutrition treatment arm differentially affected malaria infection. All models were adjusted for child characteristics (sex [binary] and age [continuous, months]), household demographics (asset index [continuous] and maternal education [categorical]), study cluster, and whether the household received the nutrition intervention (binary). Analyses controlled for arms that received nutrition interventions because they were likely confounders. 

Mediation analyses were then undertaken to assess whether anemia was a mediator along the pathway between malaria infection and being at risk for gross motor, communication, and personal social delays. The binary_mediation method in Stata [59,60,61] was employed. Like the Sobel–Goodman test for continuous outcomes, the binary_mediation method shows that mediation occurred if: 1) malaria infection was significantly associated with the mediator (anemia), 2) malaria infection was significantly associated with developmental delay in the absence of the mediator (anemia), 3) the mediator (anemia) was significantly associated with developmental delay, and 4) the effect of malaria infection on developmental delay decreased upon the addition of the mediator (anemia) to the model. The proportion of the total effect of malaria infection on the risk for gross motor, communication, and personal social delay that is mediated by anemia was also calculated and reported.

Children with missing malaria data or child development assessments were removed from analyses. Nine percent of children had missing data from one of these measurements and thus were not included in the final analyses. The final sample size was therefore 592 children. Differences between those who provided consent for blood collection and had a sufficient blood sample, and those who did not were assessed due to the low response rate and no significant differences or systematic attrition were found [36]. 

### 2.7. Ethics 

The WASH Benefits trial is registered as a clinical trial with the U.S. National Institutes of Health (https://clinicaltrials.gov/ct2/show/NCT01704105). Ethical approval was obtained from the University of California, Berkeley Center for the Protection of Human Subjects and the Kenya Medical Research Institute Ethical Review Committee. Adult participants provided written consent for themselves and their children prior to enrollment.

## 3. Results

### 3.1. Socio-Demographics and Child Health

Maternal education in this population was low: 53% of mothers did not complete primary school (Table 1). The mean age of the children was 24 months, with 29% of children stunted and 7% underweight. The prevalence of anemia among children included in these analyses was 31%, which is similar to the prevalence of anemia among children in the Nutrition intervention arm (36%) and WASH + Nutrition intervention arm (27%), and lower than the prevalence of anemia in the control group (49%) [36]. Eighteen percent of children had a positive malaria blood test suggesting that they were infected with a malaria parasite in the previous month. Of these, the majority (81%) were infected with *P. falciparum*. The percent of children that were at risk for gross motor (20%), communication (21%), and personal social (23%) delays in this population (had Z-scores in the lowest quartile based on control group domain Z-scores) were similar across domains.

### 3.2. Multivariate Associations 

Having a positive malaria test was significantly associated with increased odds of a child being at risk for gross motor (OR 2.22; 95% CI 1.35, 3.64), communication (OR 1.92; 95% CI 1.19, 3.09), and personal social (OR 2.86; 95% CI 1.81, 4.55) delays (i.e., having domain Z-scores in the lowest quartile) adjusting for child characteristics, household demographics, study cluster, and participation in the nutrition treatment arm (Model 1, Table 2). The odds of a child being at risk for developmental delays (i.e., having domain Z-scores in the lowest quartile) across all subscales decreased with the addition of anemia (Model 2, Table 2) to models, but remained significant. The strength and significance of the associations were greater for personal social delays across Models 1 and 2. Anemia was only significant in personal social analyses. Interactions between malaria and participation in the Nutrition intervention arm were not significant as the treatment arm did not play a role in this association.

### 3.3. Mediation 

Anemia was a significant mediator in the pathway between malaria infection and risk of gross motor, communication, and personal social development delays (i.e., having domain Z-scores in the lowest quartile) (Table 3). Across all subscales, malaria was significantly associated with being anemic and risk of developmental delay, anemia was significantly associated with risk of developmental delay, and the effect of malaria on risk of developmental delay decreased upon the addition of anemia to the models. The proportion of the total effect of malaria on risk of developmental delay (i.e., having domain Z-scores in the lowest quartile) that is mediated by anemia in this population was greatest with the communication subscale (16%), however, all proportions were small (Table 4).

## 4. Discussion

A positive malaria blood test was associated with increased odds of being at risk of gross motor, communication, and personal social delays (i.e., having domain Z-scores in the lowest quartile) while controlling for a range of individual- and household-level variables. The odds of personal social delays were greater than the odds of delays in the other two domains. Across subscales, the addition of the potential mediator (anemia) to models led to smaller odds of delays. Formal mediation analyses demonstrated that anemia was a mediator in the pathway between malaria infection and risk of developmental delay in all three domains. However, the proportion of the total association mediated by anemia was small.

The prevalence of malaria (18%) in this population was lower than expected given that the prevalence of malaria in the region is 42% using RDTs [62]. This could have been at least partially due to seasonality. Although studies show mixed results with varied effects based on severity of malaria and developmental domain [10,11], these findings corroborate research that demonstrates an association between malaria infection and child development, particularly among children under five years and who are not exclusively experiencing severe malaria. While few studies have undertaken formal mediation analyses, the results are comparable to two studies from Zanzibar, Tanzania, assessing malaria, hemoglobin concentrations, and motor development among children under two years [20,24]. Children with higher malaria parasite densities in Zanzibar had significantly lower hemoglobin concentrations [20]. Malaria was directly and indirectly associated with motor abilities after controlling for anemia status in these two assessments in Zanzibar [24].

This study has several strengths. The use of blood tests to indicate the presence of malaria parasites provides a robust measure of infection. Including all cases of malaria rather than only severe or cerebral incidents is beneficial for understanding associations between mild forms of malaria that are more widespread and child development. There may be a lag between infection and the manifestation of measurable effects on development, and the measurements in this study were longitudinal; however, the amount of time between the malaria blood test and the child development assessments varied. Incorporating multiple domains of child development yields a more comprehensive understanding of the implications of malaria infection. The use of a control group to calculate standardized child development scores enabled analyses to accurately assess whether a child is at risk of delay in this population. Building models by systematically adding a hypothesized mediator to analyses allowed for a thorough assessment of the potential pathways of impact. Further mediation analyses strengthened findings.

There are limitations to these analyses as the pathways between malaria and child development are complex. While there were no systematic differences found between those who provided consent for blood collection and those who did not, the low response rate could have led to selection bias. Resistance to venous blood collection was the primary reason for the low response rate, which was likely due to the high burden placed on respondents related to the collection of other biological and environmental samples, long questionnaires, and the child development assessment. The relatively small sample size could limit the generalizability and precision of the findings. Moreover, the rapid diagnostic tests detect parasites up to approximately one month after infection and do not quantify parasite load. Thus, it is difficult to determine when a child was infected and the severity of malaria, and therefore the extent to which infection could affect child development outcomes. Only one malaria test was performed and repeated infections, which may be common in this endemic area, could have larger effects on child development. Although there was time between the malaria diagnosis and child development assessment, there was only one measurement of each and therefore causal implications cannot be drawn. Further, children in the sample may have had malaria multiple times before the malaria test and child development assessment since they live in an endemic area. This could have made it more challenging to detect effects, thereby making these findings conservative. The single malaria test result is not indicative of general exposure to malaria, hence hindering interpretation of the association with the child development outcomes. Despite there being a lag between malaria and child development measurements, malaria and anemia measurements were taken at the same time, further limiting the conclusions about the pathways of impact. Finally, the mediation analysis did not yield direct and indirect effects of anemia in the complex pathway between malaria infection and the child development outcomes, simplifying the implications of the mediation findings. 

Overall, this study’s findings highlight how malaria may be associated with child development outcomes. The focus on children under five years adds to the literature as exposure to risk factors like malaria during this vulnerable time period is critical for development, yet understudied. Significant associations between malaria and gross motor, communication, and personal social domains in this young population emphasize the importance of preventing and treating malaria early in life. Patterns of malaria are likely to shift throughout Africa with climate change as certain geographies may be more impacted. Climate change coupled with human population density projections for the continent suggest that malaria transmission will remain high in the coming years [63,64], additionally highlighting the need to mitigate malaria infections. Finally, these results further inform the pathway of impact with the inclusion of robust mediation analyses demonstrating the role anemia may play. The proportion of the total effect of anemia in the pathway between malaria infection and the child development outcomes was small, suggesting that other possible mechanisms such as the symptoms of malaria infection (seizures, coma duration, psychosis, impaired consciousness, hypoglycemia, high fever, inflammation, lethargy, and compromised play/learning time), repeated infection, antimalarial medication, malnutrition, and low socioeconomic status may play a substantial role in this pathway [5,11,12,18,19]. Understanding anemia and other mechanisms in the pathway can provide further impetus for addressing malaria and inform its impacts on child development.

More longitudinal studies with repeated measures of malaria and child development outcomes can inform causal relations in addition to the timing of effects. The assessment of additional domains of development (e.g., cognitive) and long-term versus short-term effects can provide a comprehensive understanding of the impacts of malaria. The role of less severe and repeated cases of malaria can also support the targeting of interventions to the largest number of children who are most susceptible to risks for developmental delay. Longitudinal research assessing other mediators is important for better understanding the nuances of mechanisms of impact and when programs can be most effective. 

## 5. Conclusions

This study provides evidence of how malaria infection may be associated with short-term child development outcomes during a critical developmental period. These findings suggest that anemia, in this case caused by infection rather than lack of iron in the diet, may play a significant role in the pathway. The high prevalence of malaria and large percentage of children at risk for developmental delays in LMICs motivates the need for policies and programs to address the complexities of malaria infection. Preventative malaria measures and immediate treatment are imperative, particularly in light of projections of continued high malaria transmission in Africa. Overall, combating malaria may enable children to reach their full developmental potential, which can have lifelong and intergenerational benefits. 

## Figures and Tables

**Table 1:** Socio-demographic and child health characteristics of the study population (*n* = 592) ^a^.

	Mean ± SD or N (%)
Maternal education	
Some primary, not completed	314 (53)
Completed primary	151 (26)
At least some secondary	127 (21)
Child age (months)	24 ± 2
Child sex (male)	285 (48)
Stunted (LAZ/HAZ < −2)	170 (29)
Underweight (WAZ < −2)	40 (7)
Anemic (Hb < 11.0 g/dL)	186 (31)
Positive malaria test	109 (18)
At risk for gross motor delay ^b^	118 (20)
At risk for communication delay ^b^	126 (21)
At risk for personal social delay ^b^	139 (23)

^a^ An asset index was also derived by use of principal components, including housing structure, electricity, and ownership of a radio, television, mobile phone, clock, bicycle, motorcycle, gas stove, and car. ^b^ Children were considered at risk for delay if their Z-scores were below the 25th percentile of control group Z-scores for each domain.

**Table 2:** Multivariate associations ^a^ between the odds of being at risk for developmental delays ^b^ and malaria infection (Model 1) with adjustments for anemia (Model 2) as a potential mediator (*n* = 592).

	Model 1	Model 2
	OR (95% CI)	OR (95% CI)
*Gross Motor*		
Malaria (1 = positive RDT)	2.22 (1.35, 3.64) ^**^	2.03 (1.21, 3.42) ^**^
Child age (months)	0.74 (0.65, 0.83) ^**^	0.73 (0.65, 0.83) ^**^
Treatment arm (1 = nutrition)	0.71 (0.46, 1.09)	0.73 (0.47, 1.12)
Anemia (1 = anemic ^c^)		1.30 (0.81, 2.08)
*Communication*		
Malaria (1 = positive RDT)	1.92 (1.19, 3.09) ^**^	1.73 (1.05, 2.86) ^*^
Child age (months)	1.07 (0.95, 1.20)	1.07 (0.40, 0.91) ^*^
Treatment arm (1 = nutrition)	1.07 (0.71, 1.61)	1.11 (0.73, 1.67)
Anemia (1 = anemic ^c^)		1.35 (0.87, 2.10)
*Personal Social*		
Malaria (1 = positive RDT)	2.86 (1.81, 4.55) **	2.46 (1.52, 3.99) **
Child age (months)	0.82 (0.74, 0.92) **	0.82 (0.73, 0.91) **
Treatment arm (1 = nutrition)	1.15 (0.77, 1.73)	1.22 (0.81, 1.83)
Anemia (1 = anemic ^c^)		1.59 (1.03, 2.47) *

^a^ Logistic regression models, additionally controlled for child sex, asset index, maternal education, and study cluster. ^b^ Risk for developmental delay defined as having a Z-score below the 25th percentile control group Z-score. ^c^ Hb < 11.0 g/dL. ** *p* < 0.01, * *p* < 0.05.

**Table 3:** Mediation analyses of anemia in the pathway between malaria and risk of gross motor, communication, and personal social delay (*n* = 592) ^a^.

	Gross Motor ^b^ OR (95% CI)	Communication ^b^ OR (95% CI)	Personal Social ^b^ OR (95% CI)
Malaria ^c^ and anemia ^d^	4.42 (2.86, 6.82) **	4.42 (2.86, 6.82) **	4.42 (2.86, 6.82) **
Malaria and child development (no anemia)	2.15 (1.35, 3.44) **	2.04 (1.28, 3.23) **	2.91 (1.87, 4.53) **
Anemia and child development	1.46 (0.96, 2.22) ^§^	1.60 (1.06, 2.40) *	1.89 (1.28, 2.81) **
Malaria and child development (with anemia)	2.02 (1.23, 3.30) **	1.83 (1.13, 2.97) *	2.54 (1.60, 4.05) **

^a^ Table includes mediation test results of four regressions evaluating the association between: 1) malaria and anemia, 2) malaria and child development outcome (without anemia), 3) anemia and child development outcome, and 4) malaria and child development outcome (with anemia). ^b^ Risk for developmental delay defined as having a Z-score below the 25th percentile control group Z-score. ^c^ Binary, 1 = positive RDT. ^d^ Binary, 1 = anemic (Hb <11.0 g/dL). ** *p* < 0.01, * *p* < 0.05, ^§^ *p* < 0.1.

**Table 4:** Proportion of the total association of malaria on risk of gross motor, communication, and personal social delay that is mediated by anemia (*n* = 592).

	Anemia
Gross Motor	0.091
Communication	0.16
Personal Social	0.14
